# Supplementary material for: Implications for methenamine hippurate use in recurrent urinary tract infection management: Formaldehyde resistance and altered urinary composition
Source: PLoS Pathog. 2026 Mar 24;22(3):e1014081. doi: 10.1371/journal.ppat.1014081 (PMC13029678; doi:10.1371/journal.ppat.1014081)
Supplement: S1 Text — The naming of these isolates reflects their association with MH user cases A, B, C & D: All names are in the format [Case]: [Swab (S) or Urine (U)] [Month isolated]. The 11th isolate, E: S0, is shown in Fig B using a restricted formaldehyde concentration range (1–2 mM). Case E: S0 was the weakest FDHR isolate identified with respect to its MIC for formaldehyde. Fig B: Further average data of 3 independent repeats of MIC Growth assays for the case E isolate and controls identified in the screen shown in Fig 2. A concentration range of 1, 1·25, 1·5, 1·75 and 2 mM formaldehyde was used to demonstrate the growth advantage of Case E versus the FDHS control and the case B: U5 as a FDHR control isolate. Fig C: Schematic representation of the genetic architecture around frmA identified in the 11 FDHR isolates compared to the frmRAB operon and its surrounding genes in CFT073. This Fig was generated using the clinker unix software package [1]. All isolates are defined by the data represented in Fig 3 and Table 1. Clinker generated schematics are aligned to frmA for context. Table A: Comparison of components that showed a significant difference in average peak area (PA). Fig D in: Average data of 3 independent repeats for MIC Growth assays for defined strains in artificial urine at three different pH with formaldehyde added. The data shown is the average area under curve from data sets like that shown in Figs A and B. Error bars are omitted for clarity. The concentrations of formaldehyde used were 0, 0.5, 075, 1, 1.5 and 1.75 mM. This data shows that all strains respond in a similar manner showing no pH dependency to increasing concentrations of formaldehyde. Fig E: Average AUC data of 3 independent repeats for MIC Growth assays for defined strains in artificial urine at three different pH with methenamine added at T0. Error bars are omitted for clarity. The concentrations of methenamine used were 0, 0.25, 0.5, 0.75, 1.0 and 1.5 mg/ml. This data shows methenamine conversion to [file ppat.1014081.s001.docx]

**Supplementary Material**


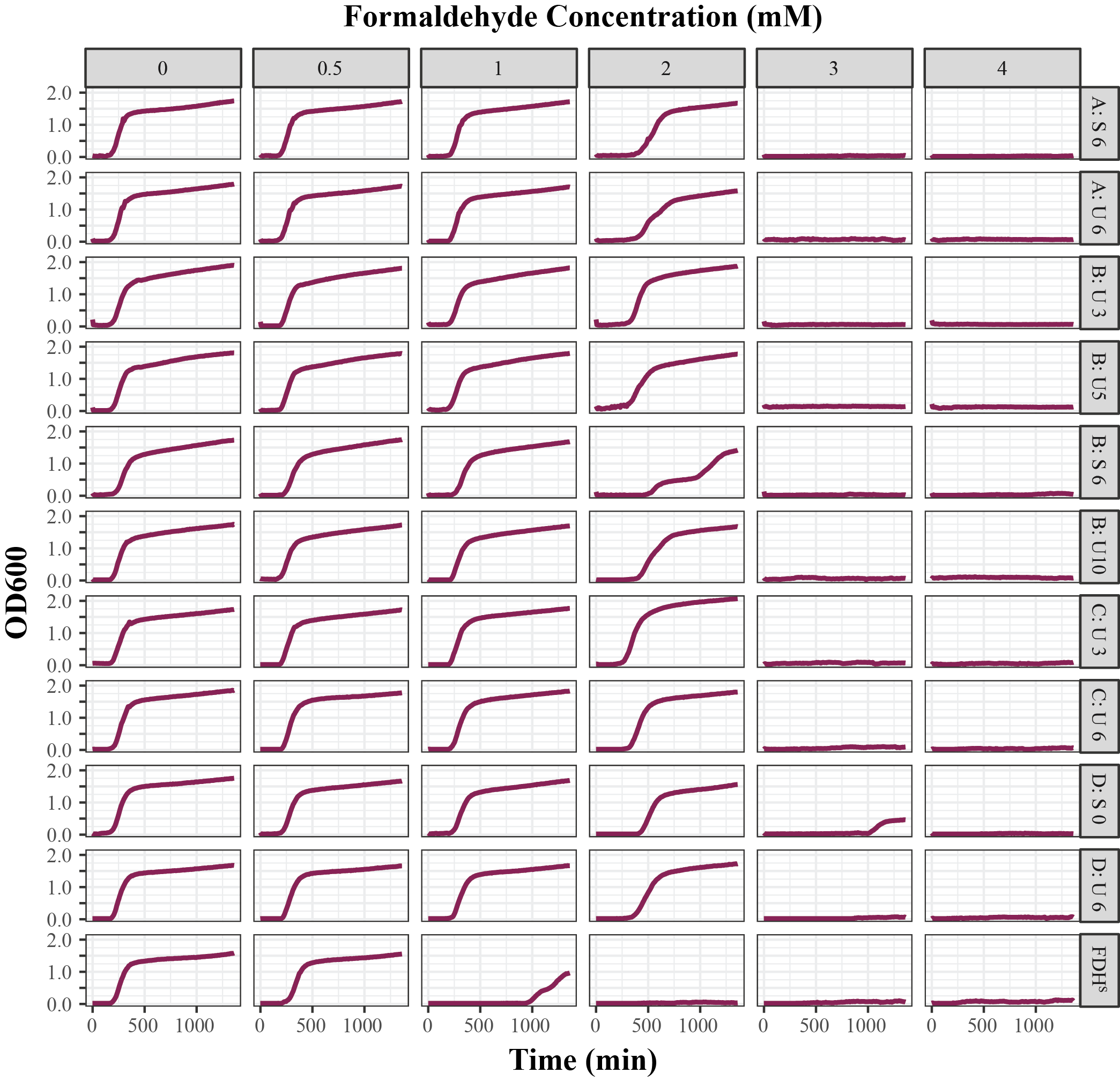


**Figure A:** Average data of 3 independent repeats of MIC Growth assays for 10 of the 11 declared isolates identified as having an MIC > 1 mM formaldehyde in the screen shown in Figure 2. The naming of these isolates reflects their association with MH user cases A, B, C & D: All names are in the format [Case]: [Swab (S) or Urine (U)] [Month isolated]. The 11^th^ isolate, E: S0, is shown in **Figure S2** using a restricted formaldehyde concentration range (1-2 mM). Case E: S0 was the weakest FDH^R^ isolate identified with respect to its MIC for formaldehyde.

**
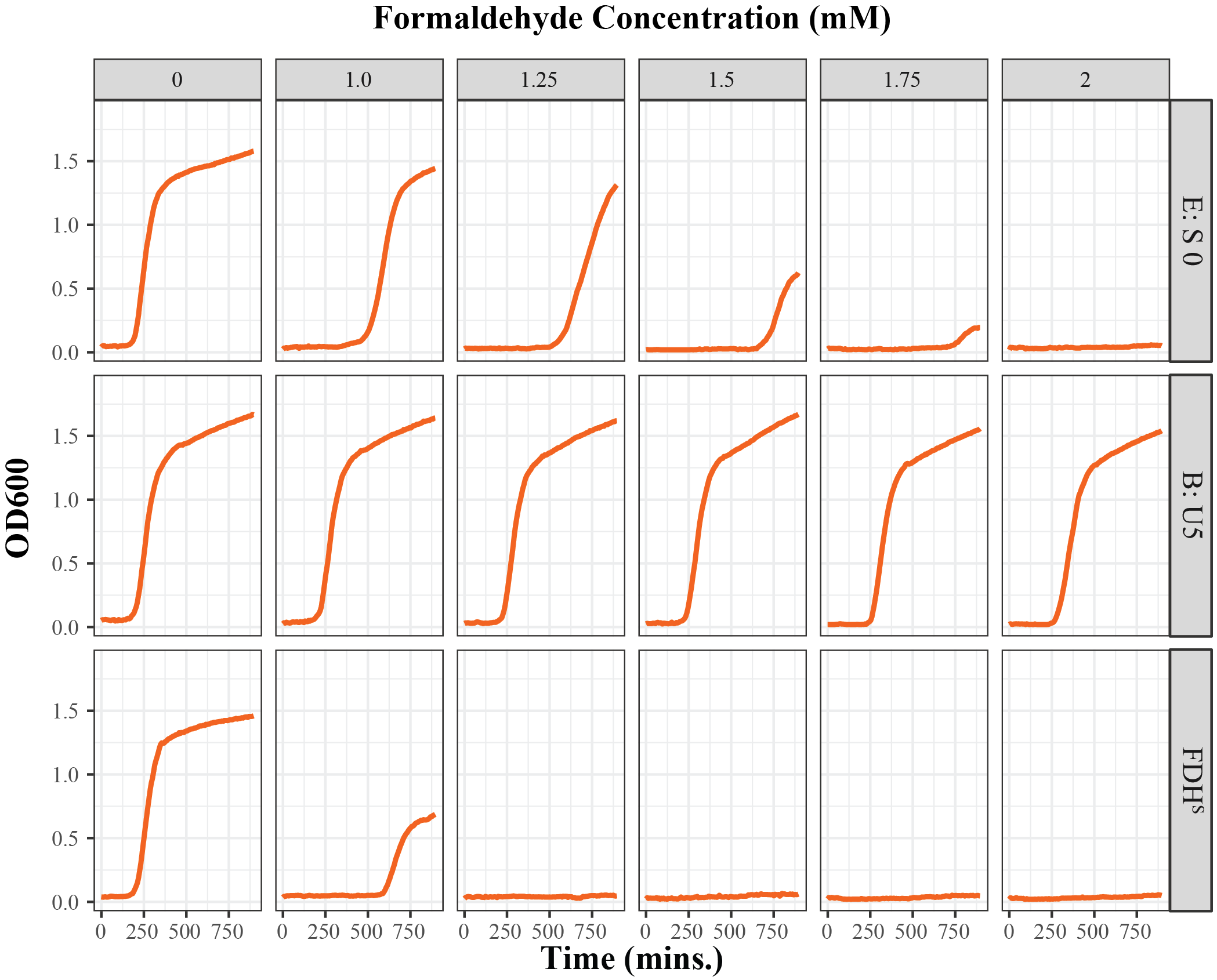
**

**Figure B:** Further average data of 3 independent repeats of MIC Growth assays for the case E isolate and controls identified in the screen shown in Figure 2. A concentration range of 1, 1·25, 1·5, 1·75 and 2 mM formaldehyde was used to demonstrate the growth advantage of Case E versus the FDH^S^ control and the case B: U5 as a FDH^R^ control isolate.


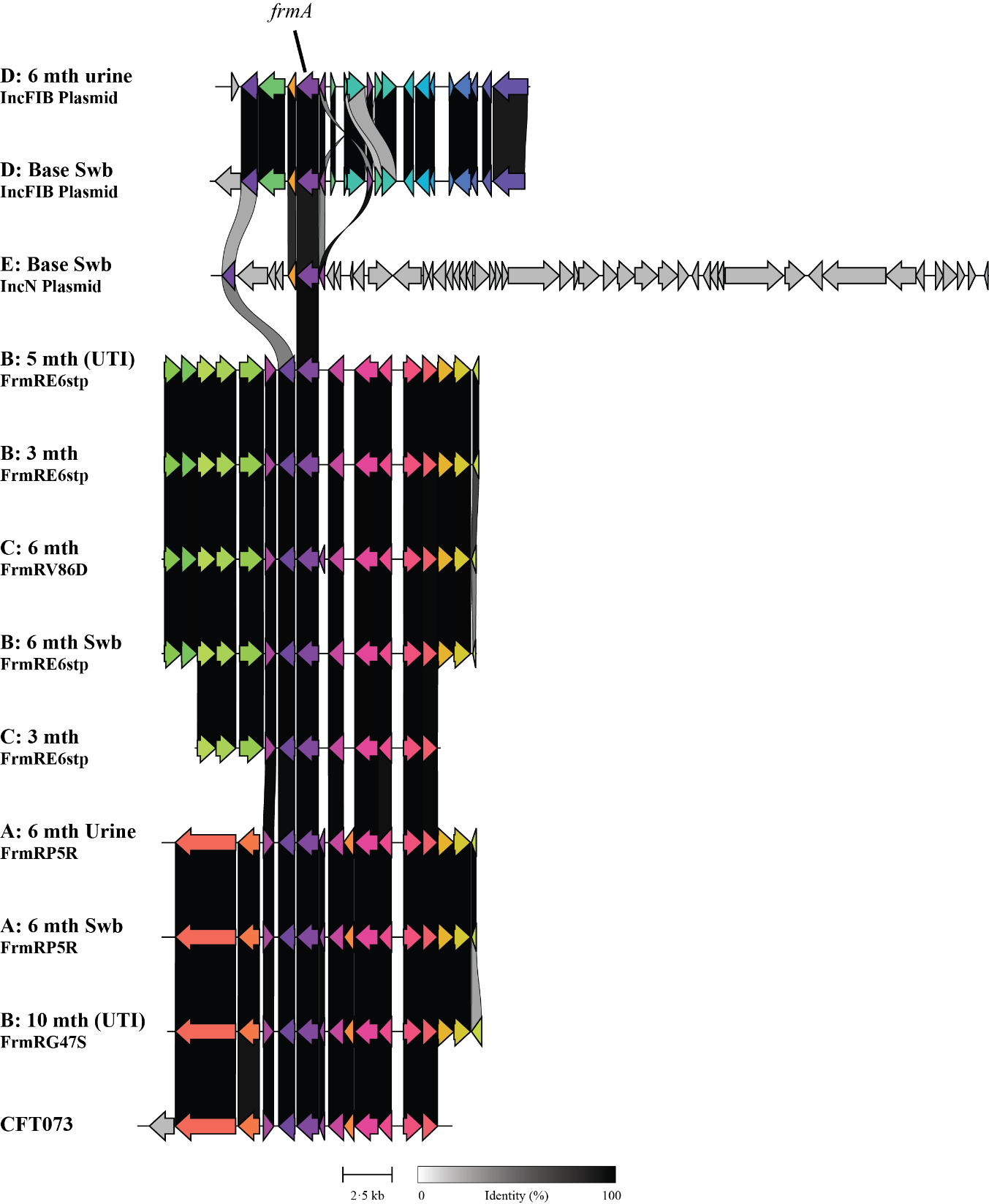


**Figure C:** Schematic representation of the genetic architecture around *frmA* identified in the 11 FDH^R^ isolates compared to the *frmRAB* operon and its surrounding genes in CFT073. This figure was generated using the clinker unix software package.[1] All isolates are defined by the data represented in Figure 3 and Table 1. Clinker generated schematics are aligned to *frmA* for context.

**Table A:** Comparison of components that showed a significant difference in average peak area (PA).

| **Trial Arm§** | **Component** | **Average PA MH** | **Average PA ABX** | **P value*** |
| --- | --- | --- | --- | --- |
| MH | CMP_A | 0.581 | 0.052 | 0.0003 |
|  | CMP_F | 0.144 | 0.058 | 0.0347 |
|  | Glucose | 0.247 | 0.063 | 0.0002 |
|  | CMP_K | 0.529 | 0.224 | 0.0000 |
|  | CMP_L | 0.033 | 0.000 | 0.0205 |
|  | CMP_M | 0.017 | 0.000 | 0.0205 |
|  | CMP_O | 18.671 | 12.980 | 0.0000 |
|  | CMP_R | 0.255 | 0.087 | 0.0009 |
|  | CMP_V | 0.111 | 0.016 | 0.0034 |
|  | Formaldehyde | 0.093 | 0.000 | 0.0000 |
|  | Formate | 0.731 | 0.507 | 0.0453 |
|  | CMP_GG | 0.016 | 0.000 | 0.0083 |
|  | CMP_HH | 0.475 | 0.000 | 0.0018 |
|  | CMP_JJ | 0.402 | 0.000 | 0.0113 |
|  | CMP_B2 | 1.696 | 0.663 | 0.0367 |
| ABX | CMP_C | 0.621 | 1.111 | 0.0033 |
|  | CMP_MM | 0.000 | 0.026 | 0.0031 |
|  | CMP_SS | 0.042 | 0.052 | 0.0427 |

§ Trial arm to which the other arm was compared to.

* Statistical test used: Mann-Whitney U Test.


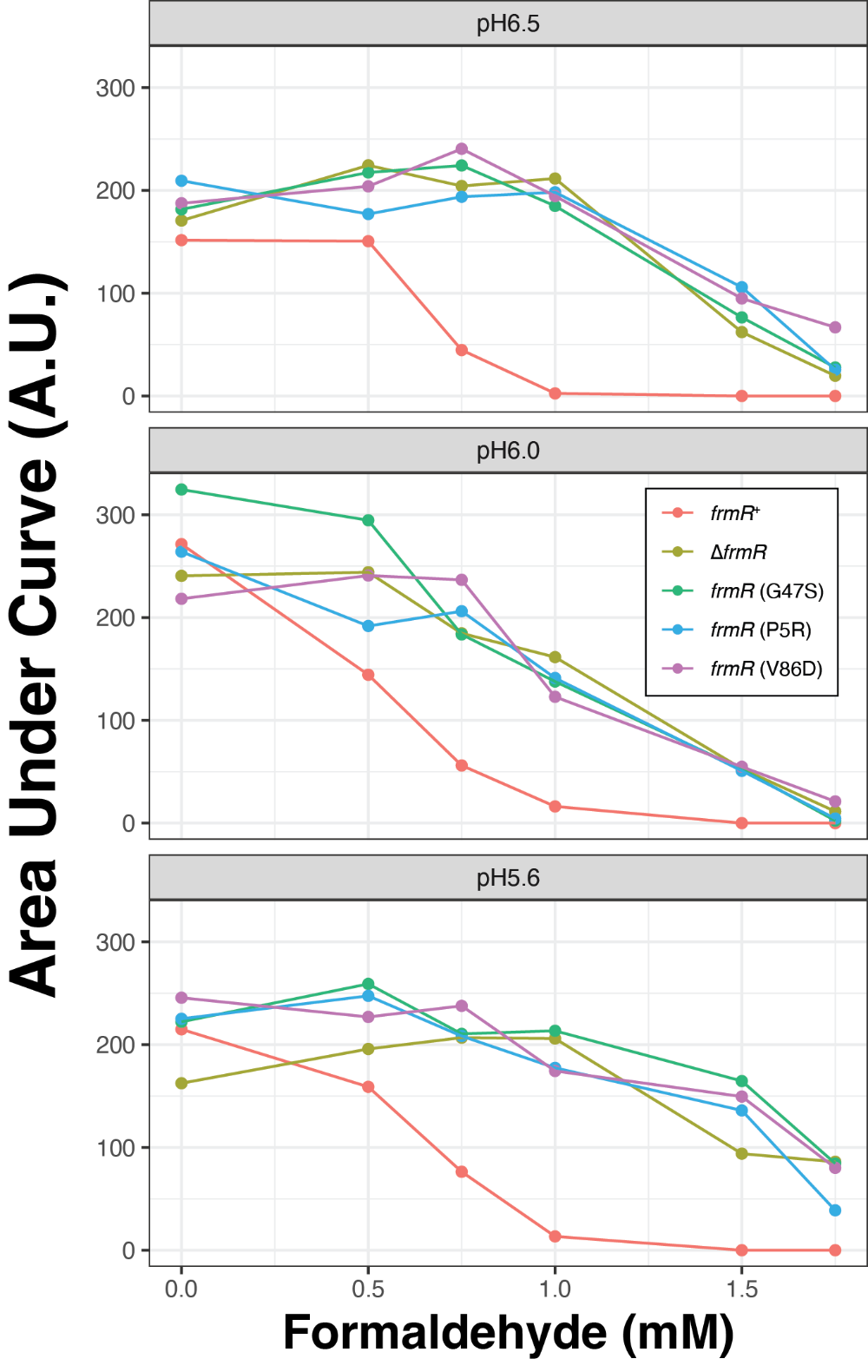


**Figure D:** Average data of 3 independent repeats for MIC Growth assays for defined strains in artificial urine at three different pH with formaldehyde added. The data shown is the average area under curve from data sets like that shown in Figures S1 and S2. Error bars are omitted for clarity. The concentrations of formaldehyde used were 0, 0.5, 075, 1, 1.5 and 1.75 mM. This data shows that all strains respond in a similar manner showing no pH dependency to increasing concentrations of formaldehyde


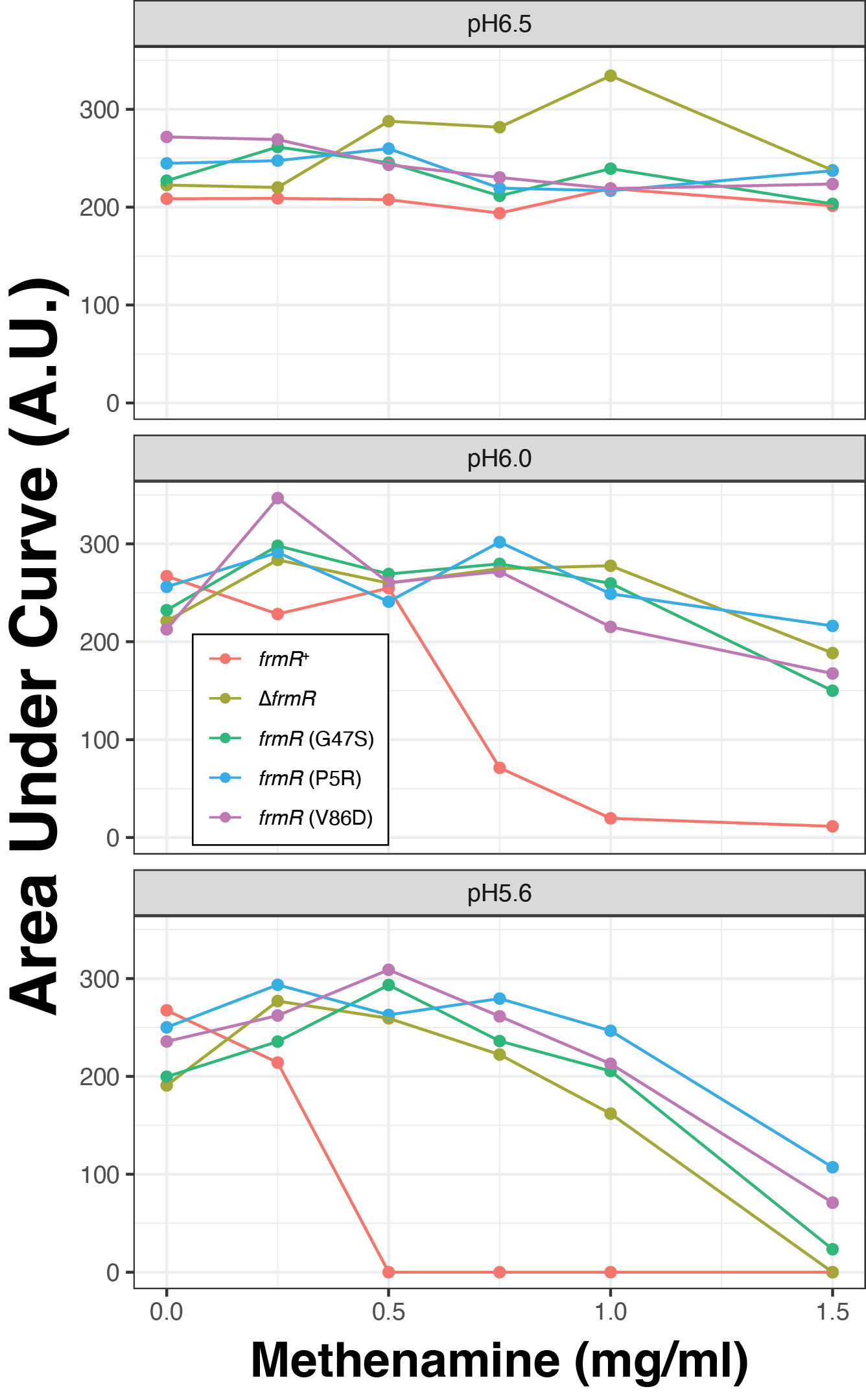


**Figure E:** Average AUC data of 3 independent repeats for MIC Growth assays for defined strains in artificial urine at three different pH with methenamine added at T0. Error bars are omitted for clarity. The concentrations of methenamine used were 0, 0.25, 0.5, 0.75, 1.0 and 1.5 mg/ml. This data shows methenamine conversion to formaldehyde is pH dependent only impacting E. coli growth at pH 6.0 and pH5.6. The strongest response to Methenamine was at pH 5.6 consistent with the pH dependent conversion of methenamine defined by Musher & Griffith (1974) [Ref 12 in main paper].


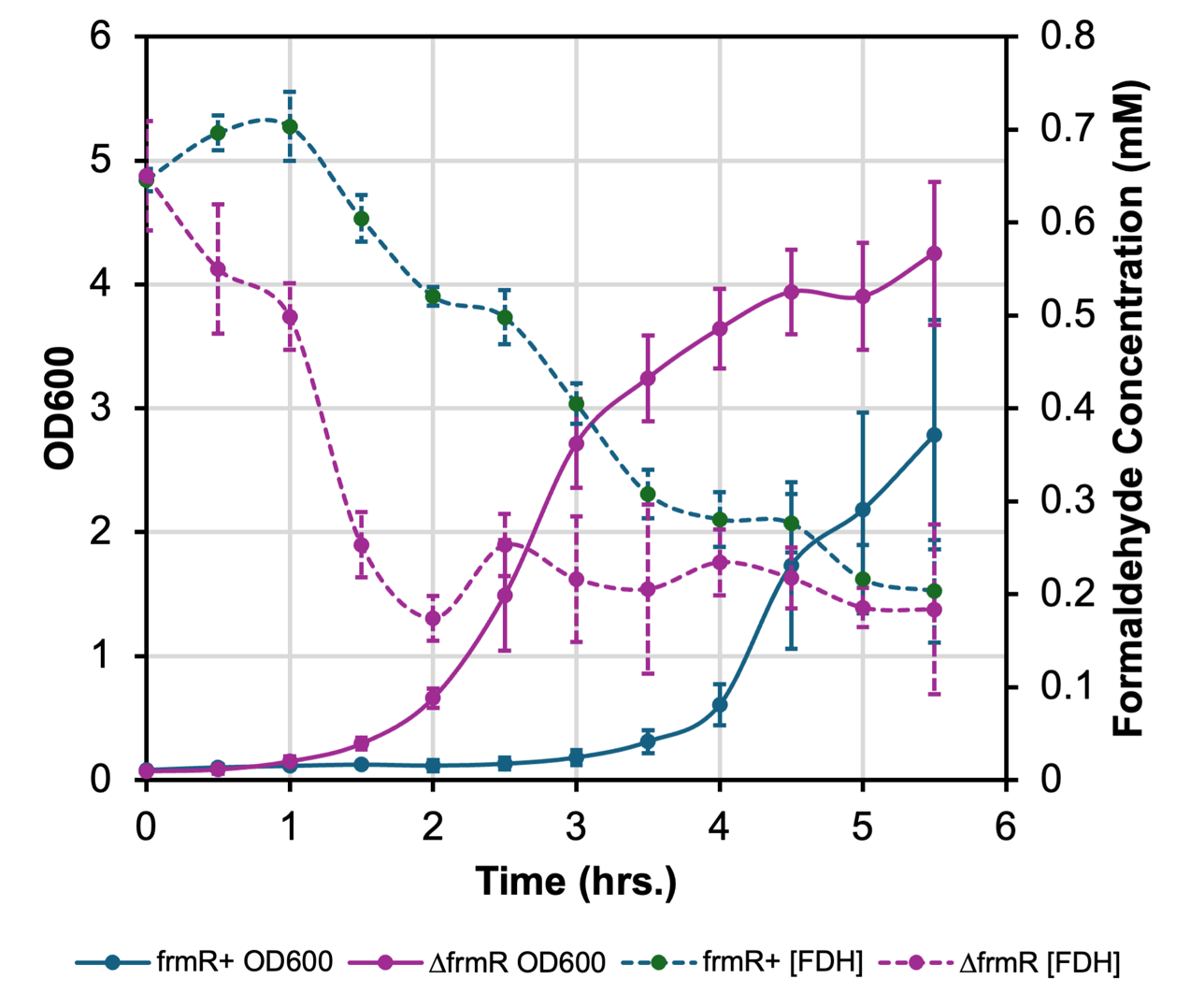


**Figure F:** Average growth of *frmR*^+^ (CFT073) and ∆*frmR* grown in the presence of 1 mM formaldehyde. Data represents that data used to derive the rate of detoxification shown in **Figure 4C**. Growth of both *frmR*^+^ and ∆*frmR* were inhibited until the formaldehyde concentration was reduced to between 0.3-0.4 mM via detoxification.

**Table B:** Clinical isolates used in formaldehyde resistance screen.

| **ID** | **contigs** | **Genome Size** | **FDH^R^ Cases** | **ENA Run Accession ID** |
| --- | --- | --- | --- | --- |
| GAT2308 | 6 | 5381408 |  | ERR14226066 |
| GAT4158 | 170 | 5128606 |  | ERR14226215 |
| GAT4512 | 6 | 5246314 |  | ERR14226063 |
| GAT4987 | 203 | 4765049 |  | ERR14226075 |
| GAT4993 | 42 | 4754255 |  | ERR14226203 |
| GAT7908 | 21 | 5407012 |  | ERR14226092 |
| GAT0245 | 35 | 5461884 |  | ERR14226061 |
| GAT1150 | 45 | 4714884 |  | ERR14226204 |
| GAT4725 | 165 | 4942605 |  | ERR14226176 |
| GAT6263 | 201 | 5205664 |  | ERR14226171 |
| GAT6726 | 255 | 5103057 |  | ERR14226184 |
| GAT8175 | 7 | 4844745 |  | ERR14226196 |
| GAT8591 | 243 | 5351193 |  | ERR14226166 |
| GAT1891 | 141 | 4825077 |  | ERR14226169 |
| GAT7073 | 3 | 5451349 |  | ERR14226103 |
| GAT1191 | 4 | 5169489 |  | ERR14226121 |
| GAT2880 | 79 | 4877576 |  | ERR14226128 |
| GAT3275 | 153 | 5085946 |  | ERR14226194 |
| GAT8334 | 114 | 5076668 |  | ERR14226116 |
| GAT9548 | 87 | 5078907 |  | ERR14226231 |
| GAT0665 | 151 | 5046956 |  | ERR14226073 |
| GAT4388 | 114 | 5114455 |  | ERR14226163 |
| GAT7924 | 215 | 5129380 |  | ERR14226232 |
| GAT9416 | 310 | 5270789 |  | ERR14226178 |
| GAT1997 | 9 | 4767085 |  | ERR14226157 |
| GAT7953 | 167 | 5104814 |  | ERR14226135 |
| GAT0206 | 9 | 5300423 |  | ERR14226148 |
| GAT0917 | 149 | 5218549 |  | ERR14226151 |
| GAT7122 | 3 | 5037629 |  | ERR14226207 |
| GAT3145 | 139 | 5479050 |  | ERR14226059 |
| GAT3148 | 76 | 4905153 |  | ERR14226210 |
| GAT3149 | 10 | 5477806 |  | ERR14226146 |
| GAT3422 | 118 | 4824080 |  | ERR14226239 |
| GAT9720 | 114 | 5186919 |  | ERR14226138 |
| GAT5933 | 5 | 5310384 |  | ERR14226078 |
| GAT2821 | 121 | 5146496 |  | ERR14226112 |
| **ID** | **contigs** | **Genome Size** | **FDH^R^ Cases** | **ENA Run Accession ID** |
| GAT2822 | 136 | 4858448 |  | ERR14226119 |
| GAT2823 | 1 | 5346020 |  | ERR14226142 |
| GAT3710 | 129 | 5332958 |  | ERR14226094 |
| GAT3711 | 234 | 4800653 |  | ERR14226223 |
| GAT7445 | 103 | 4834411 |  | ERR14226198 |
| GAT8220 | 197 | 5398390 |  | ERR14226216 |
| GAT8657 | 121 | 4949877 |  | ERR14226055 |
| GAT1068 | 69 | 4702822 |  | ERR14226064 |
| GAT4544 | 136 | 4939586 |  | ERR14226076 |
| GAT4546 | 223 | 4954806 |  | ERR14226067 |
| GAT9050 | 201 | 5078588 |  | ERR14226221 |
| GAT9054 | 16 | 5046078 |  | ERR14226205 |
| GAT4239 | 169 | 5124060 |  | ERR14226185 |
| GAT5903 | 129 | 5021869 |  | ERR14226167 |
| GAT8761 | 216 | 5237812 |  | ERR14226062 |
| GAT1476 | 121 | 4929142 |  | ERR14226057 |
| GAT6042 | 217 | 5002153 |  | ERR14226105 |
| GAT1674 | 271 | 5247569 |  | ERR14226186 |
| GAT1795 | 180 | 5437195 |  | ERR14226243 |
| GAT1018 | 206 | 5132940 |  | ERR14226172 |
| GAT5238 | 149 | 4967783 |  | ERR14226104 |
| GAT4357 | 200 | 5102287 |  | ERR14226187 |
| GAT4985 | 125 | 5014719 |  | ERR14226192 |
| GAT6549 | 7 | 5322721 |  | ERR14226195 |
| GAT8131 | 201 | 5123381 |  | ERR14226093 |
| GAT4705 | 345 | 4745574 |  | ERR14226189 |
| GAT0524 | 3 | 5255353 |  | ERR14226084 |
| GAT4131 | 91 | 5123838 |  | ERR14226227 |
| GAT8285 | 124 | 5281036 |  | ERR14226122 |
| GAT6533 | 108 | 4915616 |  | ERR14226190 |
| GAT3218 | 3 | 5218334 |  | ERR14226179 |
| GAT3970 | 35 | 5247246 |  | ERR14226110 |
| GAT5081 | 74 | 4919471 |  | ERR14226136 |
| GAT0060 | 4 | 4986035 |  | ERR14226213 |
| GAT2246 | 281 | 4954719 |  | ERR14226149 |
| GAT4413 | 225 | 5371837 |  | ERR14226152 |
| **ID** | **contigs** | **Genome Size** | **FDH^R^ Cases** | **ENA Run Accession ID** |
| GAT6402 | 78 | 5142567 |  | ERR14226208 |
| GAT8477 | 253 | 5348371 | E: S 0 | ERR14226139 |
| GAT4562 | 227 | 5074074 |  | ERR14226068 |
| GAT4573 | 3 | 5024879 |  | ERR14226143 |
| GAT2792 | 5 | 5162699 |  | ERR14226060 |
| GAT5804 | 76 | 4947009 |  | ERR14226240 |
| GAT7508 | 253 | 5325599 |  | ERR14226217 |
| GAT4971 | 120 | 5448879 |  | ERR14226090 |
| GAT7170 | 75 | 4913323 |  | ERR14226228 |
| GAT1721 | 151 | 4929242 |  | ERR14226177 |
| GAT4069 | 71 | 5234721 |  | ERR14226197 |
| GAT4431 | 223 | 5023022 |  | ERR14226123 |
| GAT7315 | 111 | 5280218 |  | ERR14226233 |
| GAT7316 | 173 | 4947718 |  | ERR14226117 |
| GAT7762 | 186 | 5193358 |  | ERR14226164 |
| GAT9264 | 92 | 5198265 |  | ERR14226129 |
| GAT4936 | 139 | 5179681 |  | ERR14226074 |
| GAT9353 | 84 | 5231152 |  | ERR14226234 |
| GAT5620 | 123 | 5117002 |  | ERR14226191 |
| GAT1355 | 34 | 4829295 | B: S 6 | ERR14226180 |
| GAT8379 | 123 | 4781313 |  | ERR14226086 |
| GAT1527 | 7 | 5212785 |  | ERR14226158 |
| GAT1528 | 122 | 5090268 |  | ERR14226058 |
| GAT5060 | 120 | 5067519 |  | ERR14226100 |
| GAT5061 | 142 | 4900532 |  | ERR14226111 |
| GAT5799 | 388 | 5248260 |  | ERR14226150 |
| GAT7745 | 169 | 4777539 |  | ERR14226106 |
| GAT2282 | 154 | 5151282 |  | ERR14226153 |
| GAT2859 | 293 | 4841409 | A: S 6 | ERR14226069 |
| GAT2860 | 154 | 4892993 |  | ERR14226229 |
| GAT4329 | 225 | 4968674 |  | ERR14226140 |
| GAT4498 | 70 | 5072614 |  | ERR14226214 |
| GAT4778 | 9 | 4752281 |  | ERR14226209 |
| GAT6345 | 1312 | 5430962 |  | ERR14226199 |
| GAT7999 | 43 | 5145816 |  | ERR14226144 |
| GAT0225 | 239 | 5058202 |  | ERR14226174 |
| GAT4858 | 120 | 4980694 |  | ERR14226244 |
| GAT6846 | 66 | 4912775 |  | ERR14226235 |
| **ID** | **contigs** | **Genome Size** | **FDH^R^ Cases** | **ENA Run Accession ID** |
| GAT6847 | 257 | 5377562 |  | ERR14226241 |
| GAT1989 | 123 | 5161162 |  | ERR14226079 |
| GAT7634 | 172 | 5211609 |  | ERR14226120 |
| GAT7635 | 6 | 5255167 |  | ERR14226211 |
| GAT9946 | 1633 | 5044925 |  | ERR14226095 |
| GAT1010 | 7 | 5110227 | D: S 0 | ERR14226113 |
| GAT2964 | 152 | 5197711 |  | ERR14226225 |
| GAT0741 | 142 | 5305688 |  | ERR14226077 |
| GAT0770 | 151 | 5971615 |  | ERR14226222 |
| GAT2279 | 61 | 5184936 |  | ERR14226131 |
| GAT6515 | 192 | 5010288 |  | ERR14226224 |
| GAT8913 | 8 | 4889599 |  | ERR14226065 |
| GAT8740 | 56 | 4926812 |  | ERR14226056 |
| GAT9733 | 5 | 5104309 |  | ERR14226170 |
| GAT1668 | 236 | 4843904 |  | ERR14226168 |
| GAT1669 | 235 | 4863217 |  | ERR14226173 |
| GAT6660 | 141 | 5171336 |  | ERR14226091 |
| GAT5741 | 104 | 8762044 |  | ERR14226130 |
| GAT4119 | 4 | 5339275 |  | ERR14226118 |
| GAT6112 | 92 | 5232590 |  | ERR14226137 |
| GAT8001 | 159 | 4924933 |  | ERR14226230 |
| GAT8502 | 88 | 5231997 |  | ERR14226154 |
| GAT3325 | 12 | 4902881 |  | ERR14226147 |
| GAT6086 | 3 | 5271891 |  | ERR14226145 |
| GAT7288 | 139 | 5251381 |  | ERR14226175 |
| GAT7766 | 78 | 5330455 |  | ERR14226236 |
| GAT8422 | 131 | 5434848 |  | ERR14226245 |
| GAT9160 | 14 | 5356849 |  | ERR14226188 |
| GAT9841 | 198 | 5356759 |  | ERR14226242 |
| GAT1991 | 128 | 4830340 |  | ERR14226080 |
| GAT1992 | 109 | 4873728 |  | ERR14226126 |
| GAT2753 | 126 | 5136565 |  | ERR14226114 |
| GAT3699 | 218 | 4957378 |  | ERR14226206 |
| GAT9506 | 312 | 5048900 |  | ERR14226193 |
| GAT8479 | 184 | 4962424 |  | ERR14226226 |
| UAT1938 | 2 | 5338195 |  | ERR14226070 |
| UAT4545 | 7 | 5197847 |  | ERR14226098 |
| UAT5523 | 3 | 5040530 |  | ERR14226081 |
| **ID** | **contigs** | **Genome Size** | **FDH^R^ Cases** | **ENA Run Accession ID** |
| UAT7588 | 4 | 5079850 | C: U 3 | ERR14226096 |
| UAT1249 | 3 | 5126658 |  | ERR14226127 |
| UAT2324 | 11 | 4932611 | D: U 6 | ERR14226115 |
| UAT8230 | 3 | 4771571 |  | ERR14226218 |
| UAT2851 | 9 | 5366830 |  | ERR14226159 |
| UAT8530 | 2 | 4696138 |  | ERR14226155 |
| UAT4823 | 4 | 4951141 | C: U 6 | ERR14226097 |
| UAT3441 | 80 | 4949926 |  | ERR14226165 |
| UAT1679 | 9 | 5306626 |  | ERR14226160 |
| UAT4171 | 9 | 4903864 |  | ERR14226161 |
| UAT5260 | 28 | 5090667 |  | ERR14226212 |
| UAT9335 | 3 | 5453417 |  | ERR14226219 |
| UAT9438 | 6 | 5091095 |  | ERR14226200 |
| UAT4828 | 4 | 5245782 |  | ERR14226107 |
| UAT7272 | 3 | 5265608 |  | ERR14226087 |
| UAT7754 | 3 | 5382325 |  | ERR14226108 |
| UAT0403 | 3 | 5176079 |  | ERR14226109 |
| UAT8654 | 12 | 5478768 |  | ERR14226085 |
| UAT7084 | 4 | 4897743 |  | ERR14226133 |
| UAT9246 | 2 | 5193278 |  | ERR14226124 |
| UAT2002 | 3 | 5113446 |  | ERR14226082 |
| UAT5722 | 8 | 5481012 | B: U 3 | ERR14226181 |
| UAT3342 | 11 | 5480226 |  | ERR14226156 |
| GAT4234 | 120 | 5416215 |  | ERR14226125 |
| UAT8548 | 3 | 5210508 | B: U 5 | ERR14226182 |
| UAT9950 | 10 | 5128370 |  | ERR14226134 |
| UAT0908 | 2 | 5361768 |  | ERR14226237 |
| UAT9332 | 6 | 4818964 |  | ERR14226088 |
| UAT1192 | 8 | 5420756 |  | ERR14226162 |
| UAT2584 | 27 | 5268602 |  | ERR14226101 |
| UAT6107 | 97 | 5078452 | A: U 6 | ERR14226071 |
| UAT6565 | 67 | 5338519 |  | ERR14226141 |
| UAT7904 | 3 | 4828311 |  | ERR14226201 |
| UAT6912 | 4 | 5275089 |  | ERR14226238 |
| UAT4066 | 91 | 5015135 |  | ERR14226099 |
| UAT5443 | 3 | 4883731 |  | ERR14226083 |
| UAT6592 | 2 | 4944992 |  | ERR14226132 |
| UAT7183 | 4 | 4969875 |  | ERR14226089 |
| UAT8354 | 1 | 4874407 |  | ERR14226102 |
| UAT9685 | 2 | 4771709 | B: U10 | ERR14226183 |
| UAT1596 | 83 | 5052705 |  | ERR14226072 |
| UAT4364 | 3 | 4806431 |  | ERR14226202 |
| UAT2019 | 3 | 4750033 |  | ERR14226220 |

**Table C:** Strains and Plasmids used or constructed in this study

| **Lab_ID** | **Strain** | **Reference** |
| --- | --- | --- |
| 23 | pKD3 / DH5a | [2] |
| 3373 | CFT073 | [3] |
| 4571 | pCas9 / DH5a | [4] |
| 4723 | pTRG-CM / c8972 | [5] |
| 4785 | pKD46 / DH5a | [2] |
| 6085 | ∆*frmR*::FRT-*cat*-FRT in CFT073 | This Study |
| 6106 | ∆*frmR* in CFT073 |  |
| 6125 | *frmR* (G47S) in CFT073 |  |
| 6137 | *frmR* (P5R) in CFT073 |  |
| 6144 | *frmR* (V86D) in CFT073 |  |

**Table D:** Primer used in this study

| **Name** | **Sequence** | **Reference** |
| --- | --- | --- |
| gmk+423-fw | CAGCGAAGAGGTCATTGC | [6] |
| gmk+599-rv | AAAGCGTCATGACGCTGC | [6] |
| rpoB+563-fw | TCGATCCGAAGGACAACC | [6] |
| rpoB+746-rv | TCACCACGCAGGCGTTCC | [6] |
| frmB-RT-F | ATGGTTGATCAGGGGTTGAG | [7] |
| frmB-RT-R | GCTCGCCAATAAAACTGGAG | [7] |
| OF513_frmA | GCAAACCATGAACACGTCTG | [8] |
| OF514_frmA | ACAGAATCACCTGGCTGGAC | [8] |
| frmR+1-KOF | ATGCCCAGTACTCCGGAAGAGAAGAAAAAGGTCCTTACTCGAGTTGTGTAGGCTGGAGCTGCTTC | This Study |
| frmR+256-KOR | CTATTTAAGATAGGCACGAACCAGTTCAATAGTGTCGTCAACGGAATGGGAATTAGCCATGGTCC |  |
| CFfrmR+660R | GGGTGGTGCCGTCTGGCATC |  |
| delFrmR+6R | GGTAATAGATTCAGCTATTTGGGCATTTCGCACCTCATCA |  |
| delFrmR+290F | TGATGAGGTGCGAAATGCCCAAATAGCTGAATCTATTACC |  |
| CFfrmR-371F | TGGTTACACGCAACTATGGC |  |

**Reference**

1. Gilchrist CLM, Chooi Y-H. clinker & clustermap.js: automatic generation of gene cluster comparison figures. Bioinformatics. 2021;37: 2473–2475. doi:10.1093/bioinformatics/btab007

2. Datsenko KA, Wanner BL. One-step inactivation of chromosomal genes in Escherichia coli K-12 using PCR products. Proc Natl Acad Sci U S A. 2000;97: 6640–6645. doi:10.1073/pnas.120163297

3. Mobley HL, Green DM, Trifillis AL, Johnson DE, Chippendale GR, Lockatell CV, et al. Pyelonephritogenic Escherichia coli and killing of cultured human renal proximal tubular epithelial cells: role of hemolysin in some strains. Infect Immun. 1990 doi/10.1128/iai.58.5.1281-1289.1990

4. Jiang Y, Chen B, Duan C, Sun B, Yang J, Yang S. Multigene Editing in the Escherichia coli Genome via the CRISPR-Cas9 System. Kelly RM, editor. Appl Environ Microbiol. 2015;81: 2506–2514. doi:10.1128/AEM.04023-14

5. Sim M, Koirala S, Picton D, Strahl H, Hoskisson PA, Rao CV, et al. Growth rate control of flagellar assembly in Escherichia coli strain RP437. Sci Rep. 2017;7: 41189. doi:10.1038/srep41189

6. Tan A, Alsenani Q, Lanz M, Birchall C, Drage LKL, Picton D, et al. Evasion of toll-like receptor recognition by Escherichia coli is mediated via population level regulation of flagellin production. Front Microbiol. 2023;14: 1093922. doi:10.3389/fmicb.2023.1093922

7. Gonzalez CF, Proudfoot M, Brown G, Korniyenko Y, Mori H, Savchenko AV, et al. Molecular Basis of Formaldehyde Detoxification: CHARACTERIZATION OF TWO S-FORMYLGLUTATHIONE HYDROLASES FROM ESCHERICHIA COLI, FrmB AND YeiG *. J Biol Chem. 2006;281: 14514–14522. doi:10.1074/jbc.M600996200

8. Herring CD, Blattner FR. Global Transcriptional Effects of a Suppressor tRNA and the Inactivation of the Regulator *frmR*. J Bacteriol. 2004;186: 6714–6720. doi:10.1128/JB.186.20.6714-6720.2004
