# Supplementary material for: Implications for methenamine hippurate use in recurrent urinary tract infection management: Formaldehyde resistance and altered urinary composition
Source: PLoS Pathog. 2026 Mar 24;22(3):e1014081. doi: 10.1371/journal.ppat.1014081 (PMC13029678; doi:10.1371/journal.ppat.1014081)
Supplement: Attachment — Submitted filename: PPATHOGENS_ReButtal.docx [file ppat.1014081.s002.docx]

**Reviewer #1:**

This paper takes on a fascinating question: does an “antiseptic” lead to resistance in a treatment setting. Methenamine isn’t an antibiotic, but it is converted to the toxic molecule, formaldehyde, when the pH is sufficiently low. While I find the idea that this paper argues against to be incredibly naïve – that only antibiotics generate resistance – I am thrilled to see someone address this. They clearly show that there are formaldehyde resistant UTI strains of E. coli and that those alleles contribute to survival with MH at low pH. Overall I think what they did was fairly solid.

We would like to thank Reviewer #1 for a such a positive summary of our work.

Reviewer #1: Very naïve discussion of formaldehyde. This is probably the biggest weakness. Throughout there are many omissions and some errors. There is actually a pretty decent literature base in the roles of aldehydes in pathogens. This has even led to an “aldehyde hypothesis” that cells generate these to help kill pathogens. I have pasted in a few references below that can serve as a starting place for this

With the above statement from reviewer #1 they have provided us with a good collection of citations to reflect on. Our summary of this reviewer’s comments was that we had:

a) Neglected to introduce the impact of formaldehyde sufficiently in the introduction
b) Omitted several points of discussion relating to the formaldehyde literature during our discussion.

To address this, we have now expanded on our introduction to formaldehyde and used the comments of reviewer #1 to strengthen given points we make in the discussion.

Second, there is a fair amount more about E. coli and formaldehyde out there. Some of it is directly related (see the heterogeneity in resistance coming from higher frmA in the first paper) and other points to distinct mechanisms to explore (thioproline in the 2nd). In the first paper, they find an identical behavior: the timing of E. coli recovery was set by having to reduce the formaldehyde added to below a threshold.

We have now correlated our findings on E. coli growth response to formaldehyde exposure to Jordan et al (2022) in the discussion.

Third, evolution to increased formaldehyde has been done in several organisms. The results are not straight-forward. Putting in “evolution of formaldehyde resistance” into google pulls up a paper (below) using a methanol eating bug, Methylobacterium, where the mutations were found to be in novel sensors and NOT in formaldehyde oxidation. The major point here is that you -could- have found mechanisms other than the known formaldehyde oxidation pathway as the beneficial mutation in your evolved strains, but you didn’t.

We have included a discussion point relating to this and the *efaG* story.

In discussing the E. coli pathway, FrmA is not an oxygenase. No molecular oxygen is involved. It is an S-hydroxymethyl-GSH dehydrogenase that is NAD-dependent (generating NADH). It is also important to mention that formaldehyde reacts with reduced glutathione (often stated as GSH) and not with the oxidized form (written GSSG). When the formyl group is hydrolyzed, it is recycled as GSH (thus no net reduction is needed to regenerate the cofactor).

This has been corrected in the introduction

Another point of concern is the effect of pH on the reactivity of formaldehyde. Low pH is needed to break down methenamine. There is, however, a second effect. At low and high pH formaldehyde is also less reactive with molecules such as the nitrogen groups of pterins. I have seen this demonstrated in the following paper. Because of this, E. coli is known to reduce the cellular pH when aldehydes (like methylglyoxal) build-up by using the Kef efflux pumps (many papers from Ian Booth’s lab).

We now cite the proposed paper in the discussion when discussing future directions of research. We agree pH may play an as yet fully appreciated role in inhibiting E. coli growth during MH use. Although, the data in Figure S4 does not strictly correlate with the findings reviewer #1 points to in the Vorholt et al (2000) paper as we see similar inhibition at all pHs used.

It is great to see a lab phenotype for the frmR deletion strain. However, this was only a single hour of lag. And the patients were treated for a year. Is that a big enough difference? I think that needs some substantial discussion, for it isn’t clear to me that there would be very strong selection then. Most likely, the lab is not the same as a patient, of course, but this needs to be addressed.

Yes, there is strong evidence in the literature that there are good and bad E. coli bladder colonisers and that E. coli requires a given growth rate to maintain a population in the bladder. We cite this work and have expanded our discussion on this point.

Reviewer #1: p.5, line 112 – Here and elsewhere, the S in S-hydroxymethylglutathione (and other similar compounds) should be italicized.

Done

p.7, line 132 – The urine concentration is stated as ~1 millimolar and then the numbers listed below are 0.88 to 122.1 micromolar. Are both of these units correct?

Yes, the units are correct. However, to reduce confusion when discussing µM ranges we have now converted all units in such sections to µM, whereas elsewhere we have left this as mM and µM. We have made it clear at several points during our manuscript that a limitation is the retrospective format of this secondary analysis has the potential to impact formaldhyde detection. However, even taking this into account our detection correlates to previous work.

p.11, line 191 – Correct “overtime” to “over time”

Done

p.12, line 210 – If the loci on the plasmids are also frmRAB, as I believe is being stated, then they would be called redundant (homologous extra copies) rather than degenerate (non-homologous, alternative pathways to accomplish the same task).

We have reworded this statement to reflect more what we observe in Figure S3. Both plasmids encode frmA and frmB homologus but not in the same operon structure as observed for the chromosomal locus.

p.12, line 211 – For the substitutions, it is hard to read them as FrmRE6stp. I would change all of these to just E6stp, etc., since they all occur in the same protein (FrmR). They are much easier to understand that style in table 1.

We have adjusted this to as suggested in the main body of the text and figure 4.

p.13, line 226 – Relevant to the point made a couple points above, rather than how similar the adhC is to a plasmid, it would be most helpful to know how similar they are to the chromosomal frmRAB cluster. Might even want to turn to AA identity or similarity if they are very different.

We have adjusted the text to reflect the comparison of both DNA and amino acid sequence in this section.

**Reviewer #2:**

This submission is a secondary analysis of both urine samples and E. coli isolates obtained those samples (and from perianal swabs) during the ALTAR study., which compared symptom outcome in adult females with recurrent UTI who were treated prophylactically with either methenamine hippurate (MH) or with one of a number of antibiotics. One finding of the ALTAR study was “...a marginally higher rate of UTI episodes in those taking MH compared to the prophylactic antibiotic group. The goal of the authors was “...to understand what factors could contribute to this elevated risk of a UTI during MH use compared to daily low dose antibiotics.” The authors found that a few E. coli isolates obtained from a small subset of the participants exhibited substantially elevated minimal inhibitory concentrations for formaldehyde. Some of these formaldehyde-resistant (FDHR) isolates had inactivating mutations in the gene that encodes FrmR, the repressor protein for the frmAB operon, which encodes the formaldehyde detoxification system. Other isolates carried plasmids that encoded homologs of frmAB. This finding of elevated resistance is significant for two reasons. (1) The proposed mode of action for MH is its dissociation in the urinary tract into methenamine and hippuric acid. The hippuric acid reduces the urine pH, which leads to dissociation of the methenamine into formaldehyde and ammonia. It is the formaldehyde that is thought to inhibit bacterial growth. (2) MH is considered to be an antiseptic and thus not subject to development of resistance. The authors discovery that E. coli can evolve increased resistance (elevated MIC) requires a reevaluation of the risks associated with prophylactic MH treatment. The finding of these FHDR isolates led the authors to investigate a number of parameters associated with these isolates, MH treatment, and formaldehyde concentrations.

I consider the science to be rigorous and have absolutely no problems with it. My issues are all about the writing. The Abstract is terribly confusing. I had to read the Results to understand what the authors were trying to say in the Abstract. This is a secondary analysis, which the authors do not explicitly state. Because the Methods come after the Discussion, important details necessary for the reader to understand the Results are not provided. The text for some figures is not written in a simple intuitive fashion. The upshot is that it took me a lot longer to follow the science than it should have.

We would like to thank Reviewer #2 for such a positive and constructive review of our manuscript. We agree with reviewer #2 that the suggested edits and additions have strengthened our narrative. Due to the nature of these suggestions, we have been able to incorporate the majority into the revised manuscript.

Issues with the Abstract

Lines 33 - 38. These sentences are confusing. They only became clear after I read the Results. The authors state that E. coli uses the frmRAB operon to detoxify formaldehyde and that, unexpectedly, a small percentage of isolates were able to grow in the presence of formaldehyde. They tell us that some fmrR alleles encoded non-functional variants and that others carried plasmids that encode frmA homologs. But the reader cannot understand the significance of these findings without some information concerning the roles of FrmR and FrmAB. It turns out that FrmR is the repressor for the operon. If it is non-functional, then the operon is expressed constitutively, leading to more detoxification of formaldehyde. FrmA is an enzyme involved in the detoxification. The authors must rewrite this part of the abstract, so it makes immediate sense to the reader.

Line 40. The urine composition was altered in what way? A little more detail would be helpful, especially since 2 of the alterations were formaldehyde and formate, and another was glucose. Furthermore, the authors should mention that formate is the result of formaldehyde detoxification.

We have re-written the abstract taking on board these constructive comments.

Issues concerning Methods

Line 135. Since the methods are after the Discussion, the Results must explain the original ALTAR study design sufficiently for the reader to understand the Results including the following:

1) First, the authors should state explicitly that this is a secondary analysis of the original ALTAR study.

2) It is reasonable to expect the authors to tell us upfront that there were 2 arms to the study (MH and ABX), that the antibiotic treatment was prophylactic in nature, and that several different antibiotics were prescribed in the ABX arm. At least then the reader won’t be surprised to read about a participant on Nitrofurantoin on line 172.

3) The duration of treatment.

4) The duration of the sample collection.

5) That both urine samples and perianal swabs were obtained. Otherwise, lines 179-180 come as a complete surprise. See comment above.

6) The collection method of the urine, presumably the midstream voided method.

7) The number of participants in each arm and how many of those were analyzed in this secondary analysis. Don't expect the reader to count the numbers in panel B.

8) Whether there was any significant difference in any demographic or symptomatic variable between the 2 arms. I don't expect a table of the participants, but it is important to know that the ALTAR study did or did not find any significant differences between the participants in the 2 arms.

We have now included a summary of ALTAR at the beginning of the results as requested. In doing so we have also made it clear that this is a secondary analysis of the samples that fit the declared criteria.

Line 159. This comes as a complete surprise. What isolates? How were they isolated? They were sequenced? I'm not asking for the methods here. I'm asking that you state that you isolated E. coli somehow and that you sequenced their genomes. Then tell us that you screened them.

This has been adjusted but also the introductory summary of ALTAR at the beginning of the result now allows the reader to make sense of this section.

Issues with figures & tables.

I like Figure 1. It is well designed and quite intuitive. Ditto for Figure 2.

Figure 3. This figure is either not intuitive or not well described. I get what the authors are trying to say but it took me a while to understand all of the parts of the figure. More explanation would be helpful in the text and in the legend.

Figure 3 legend has been re-written and more information included both in the legend and the main body text.

More Figure 3. What do the circles and squares mean? And on line 199, the authors say “see legend.” This is the legend. You mean the information in panel A. I would say “see Panel A.”

This suggestion has been included in the re-written legend.

Line 218. I think the phrase "order of cases" is confusing. I think you mean that they refer to the cases shown in Figure 3. It's the word "order" that is problematic.

This has been removed and we have used the above comments with respect to figure 3 to clearly define cases A-E in the main body of the text.

Figure 4A. Why is this figure oriented the opposite of the previous growth curves?

We have adjusted figure 4 to reflect the format of figures S1 and S2. We have also incorporated reviewer #1’s suggestion to omit “FrmR” when discussing the variants.

Line 317: Figure 6. Same problem as above. "See legend" is confusing because this is the legend. Maybe the best way is to say something like "See upper left panel."

This has been adjusted

Figure S1. The legend should say that these are 10 of the 11 resistant isolates so the reader understands the statement about isolate E: S0.

This has been adjusted

Fig. S1. The legend says that Case E: S0 is shown in Figure S3. It is actually S2.

This has been adjusted

Figure S3 legend. What is "the clinker?" Is it a bioinformatic tool? The problem is the word "the." Delete it in this sentence and in the last one.

This has been adjusted

Also, the reference 8 is not in the bibliography.

Apologies for this oversight, this has been adjusted

Issues concerning dogma

Lines 33, 110, 157. Concerning the use of the phrases “uropathogen E. coli” and “major uropathogen E. coli.” I don't want to pontificate but there are 2 issues with these phrases. First, there are no obvious differences between the supposed UPEC strains and their closest relatives. What appears to be more important is the phylogroup of the isolate. Second, there’s good reason to support Arturo Casadevall’s contention that the more appropriate term is “bacterium with pathogenic potential.” Pathogenesis requires a host; therefore, what does it mean to call a bacterium a pathogen? The way I’ve been handling these issues is to use the term pathogenic potential and to call CFT073, UTI89 and NU14 classic UPEC strains.

We fully agree with this statement and respect that it is taking time for the field to alter how we describe E. coli and its association with the urinary tract. We have therefore followed this suggestion and removed the term uropathogen where necessary, while defining CFT073 as a classic model UPEC strain.

Line 76: This is old dogma. It assumes that dysbiosis is not a cause. One doesn't need to say "invading." Delete it.

Done

Comments concerning the Discussion

The authors appear to be unaware of a relatively recent paper that shows that methenamine treatment does not necessarily eliminate pathogens (Khan et al., 2025 PMID: 41317299). Instead, it alters urinary microbiome composition. This is likely related to the authors observation that treatment with MH alters the composition of the urine. The authors might want to read the Khan paper and comment on it in their Discussion. It’s relevant.

This is a bit harsh since the publication date of the Kahn paper was 1.5 months after the submission of this manuscript. However, we fully agree that within the process of revising this manuscript we can now include the correlation of our HPLC analysis to that of the findings presented in	Khan et al (2025).

Reviewer #2: Other comments:

Line 36. The proper word is "unexpectedly" not “unexpectantly.”

This has been adjusted

Line 39. The authors should tell the reader that CFT073 is a classic UPEC strain.

This has been adjusted

Line 42. Alleles do not grow. Isolates that encode alleles grow.

In re-writing the abstract, this has been adjusted

Line 96. Define NICE.

This has been adjusted

Line 97. The authors define the acronym MH on line 101 but it should be defined on line 97 when "methenamine hippurate" is first mentioned.

This has been adjusted

Line 121: Rate is a function of time, which the next sentence clarifies. But, this must read UTUI episodes per year.

This has been adjusted

Line 122. The authors c/should add that this could be statistically and biologically significant - i.e. development of resistance.

We have tried to include this statement.

Lines 138-140. This statement should be made in the Discussion, not the Results. It is opinion/inference/hypothesis not results.

This has been adjusted

Line 142. "These data...." Data are plural.

This has been adjusted

Line 160. The reader does not know why you were surprised. Why were you surprised? Use your answer to write a transition phrase. Something like "Because X, we were surprised...."

Good point, this has been adjusted

Line 161. Would be helpful to state what these elevated MIC were.

We have now included a summary of the identified MICs at this point, thank you for this suggestion.

Line 225. This sentence is ambiguous. Lack of sequence data of which - pVU3695 or the ALTAR plasmids? It only becomes clear when you read the next sentence.

This has been adjusted in reflection of both reviewer’s comments regarding this paragraph.

Line 227. Again, this is ambiguous until you read the end of the sentence. Be explicit.

This has been adjusted

Line 230. But you don't have the full sequence of the plasmid. Is this 88% to adhC (frmA)? Be explicit.

We have changed the narrative of this section in response to both reviewers by now declaring both the similarity for DNA and coding sequence.

Line 259. "Compliment" means to praise. The proper term is "complement." Sorry English can be a pain sometimes.

This has been adjusted

Line 296. This whole section is confusing. It’s the writing not the data. It took me a while to figure out what the authors were trying to say. The show 2 different types of data for 2 different strains (WT and mutant) at 3 different pHs. And they jump immediately to the outlier, which is - of course - the interesting information. The sentence on line 299 seems odd since the authors just said that they measured formaldehyde concentration. It would be better if they added the information that they measured growth in the previous sentence. Then it makes sense to talk about inhibition of growth. But I recommend that the authors mention the results of the higher pHs first, then talk about what happens at the lower pH.

We have adjusted this section in line with this suggestion. We agree in doing so the data becomes easier to follow.

Line 305. This sentence is not intuitive. Do you mean that WT growth was inhibited as long as the FDH remained above 0.4 mM? And that there was lag until the concentration remained above that concentration?

This has been adjusted

Lines 330, 340. Remind the readers that FrmR is a represssor.

This has been adjusted

Lines 426. Please describe the collection methods for both urines and swabs. After mentioning the urines and swabs at the beginning of the Results, the details should be included here in the Methods.

This has been adjusted with reference to the published study protocol of ALTAR.

Line 427. From "from fresh urine" correct?

Yes this was from fresh urine as all samples were processed through the local Microbiology units.

Issue of simple curiosity

Line 118. Interesting result. If the methenamine and hippuric acid dissociate in the kidneys, then what is the mode of action in the gut?

From our own understanding of the literature there is no assumed breakdown of methenamine in the gut. However, this is an understudied area that the work reviewer #2 has been involved in with Kahn et al (2025) and our own findings should provide a foundation for future studies to investigate this impact.
